# The relationship between mindfulness, self-control, and short video addiction among medical students

**DOI:** 10.3389/fpsyg.2026.1746374

**Published:** 2026-05-01

**Authors:** Yuting Luo, Yulian Li, Quanmin Wu, Pengchong Shi

**Affiliations:** 1 The School of Nursing, Fujian Medical University, Fuzhou, Fujian, China; 2 Clinical Skills Center, Fujian Medical University, Fuzhou, Fujian, China; 3 Department of Clinical Laboratory, Fujian Medical University Union Hospital, Fuzhou, China

**Keywords:** college student, latent profile analysis, mindfulness, self-control, short video addiction

## Abstract

**Background:**

The prevalence of short video addiction is rising among college students, potentially exerting detrimental effects on their physical and mental wellbeing. Medical students, who experience relatively higher levels of academic stress, may be particularly susceptible to the adverse consequence of short video addiction. However, the specific factors influencing this addiction and the underlying mechanisms remain insufficiently understood.

**Objective:**

This study aims to examine the relationship between mindfulness, self-control, and short video addiction, as well as to identify potential profile characteristics of short video addiction among medical students.

**Methods:**

The study employed the Mindful Attention Awareness Scale, the Self-Control Scale, and the Short Video Addiction Scale. A combination of variable-centered and person-centered research methodologies was utilized. A total of 892 medical students participated in the survey.

**Results:**

The results revealed the following: (1) Mindfulness and self-control abilities were found to be negatively correlated with short video addiction. Self-control served as a mediating factor between mindfulness awareness and short video addiction, exhibiting a total indirect effect of 83.33%. (2) Short video addiction was categorized into three levels: low-risk addiction (17.60%), medium-risk addiction (57.06%), and high-risk addiction (25.34%). (3) Logistic regression analysis revealed that students with low self-control were more likely to fall into the medium-risk and high-risk addiction categories. Additionally, non-only children were more likely to be classified into the medium or high-risk addiction groups compared to only children. (4) The mediation analysis based on latent profile categories shows that self-control in the medium-risk addiction group plays a mediating role between mindfulness and short-video addiction.

**Conclusion:**

This study underscores the significant role of mindfulness and self-control in the intervention and prevention among different populations at risk of short video addiction. It also highlights the impact of individual demographic characteristics on the occurrence and progression of short video addiction. The implications of the research findings and the limitations of the study are thoroughly discussed.

## 1 Introduction

According to the “56th Statistical Report on the Development of China's Internet,” as of June 2025, the number of internet users in China reached 1.123 billion, with an average weekly online time of 30.6 h per user. The number of internet video users stood at 1.085 billion, of which short video users accounted for 1.068 billion, representing 95.1% of the total internet user base. Short videos are defined as online videos published and shared by various new media, typically lasting from a few seconds to several minutes (Dong et al., 2023). These videos are characterized by their fragmented nature, rapid pacing, strong interactivity, and diverse content. The underlying algorithms analyze users' preferences to deliver customized content, which can restrict users to a narrow information flow, resulting in passive information consumption and increased dependence on the recommended content (Huang et al., 2023). Consequently, short video addiction (SVA) has emerged in recent years as a subset of internet addiction and mobile social addiction. This study defines short video addiction as a chronic or periodic obsession stemming from repeated use of short video applications, leading to significant psychological and behavioral needs and dependencies (Wu et al., 2024; Qin et al., 2019). Research indicates that the addiction and dependence rates among college students for short videos range from 21.63% to 31.99% (Li et al., 2021b). This excessive and prolonged use of mobile applications has adversely affected the physical, psychological, and cognitive health of young individuals, particularly college students. For instance, it can easily distract students, hinder academic progress, contribute to academic procrastination and burnout, and exacerbate feelings of anxiety and depression (Feng et al., 2025). Furthermore, excessive use of social media may negatively impact the sleep quality of college students, resulting in attention deficits and potentially harmful effects on brain neural development (Wang et al., 2021). Medical students, as a distinct subset of college students, are particularly susceptible to stress, which has been linked to short-video addiction (Zhan and Zhu, 2025). Consequently, they may exhibit a higher propensity for developing such addictions (Zhan and Zhu, 2025). A recent study indicates that the SVA rate among nursing undergraduates in China is as high as 30.2% (Guo et al., 2023), significantly exceeding the 21.6% rate observed in the general college student population in China (Li et al., 2021b). As vital future professionals in the healthcare sector, the mental health and behavioral patterns of medical students profoundly influence the healthcare system in our country. Research has demonstrated that problematic smartphone use can divert medical students' attention during internships, potentially leading to avoidable medical errors (Zarandona et al., 2019). Furthermore, the capacity to manage work efficiently is a crucial professional attribute for medical students in their careers. The establishment of procrastination habits may hinder their ability to deliver high-quality medical services to patients and could jeopardize patient safety. Therefore, investigating the phenomenon of short-video addiction among medical students and its underlying mechanisms is essential for developing targeted intervention strategies and fostering a healthy digital environment to mitigate this addiction within this demographic.

### 1.1 The mindfulness and short-video addiction

Mindfulness refers to a state of consciousness that emerges by directing attention toward the present goal, as well as the non-judgmental acceptance of various experiences at this moment (Kabat-Zinn, 2003). Currently, most research primarily focuses on the intervention of mindfulness in addictive behaviors, such as mobile phone addiction, while a smaller portion of research examines the relationship between mindfulness and short-video addiction. Existing studies have found that college students with a higher level of mindfulness tend to exhibit lower tendencies toward mobile phone addiction (Kim et al., 2018). Furthermore, mindfulness may act as a mediator between college students' short-video addiction and academic participation, as students with a higher degree of short-video addiction show lower levels of mindfulness and reduced academic participation (Li et al., 2024). A substantial body of research indicates that mindfulness is associated with a reduction in addictive behaviors (Parisi et al., 2023; Bowen and Vieten, 2012). Based on the definition of mindfulness (Kabat-Zinn, 2003; Chen et al., 2021), its effectiveness in addressing addictive behaviors may stem from the ability to accept the negative emotions and thoughts triggered by these behaviors. This acceptance can potentially diminish the relationship between negative emotions and addictive behaviors, ultimately alleviating such behaviors (Witkiewitz and Bowen, 2010; Witkiewitz et al., 2012). Additionally, existing research has found that individuals prone to short-video addiction display impaired performance in attention tasks (Chen et al., 2023; Qin et al., 2022; Ye et al., 2022). The increasing prevalence of mobile short-video addiction is significantly negatively correlated with the neural activity associated with attention functions in the theta frequency band of the brain (Yan et al., 2024). Besides, research on meditation has established connections between theta and alpha oscillations and states of internally directed attention as well as positive emotional experiences (Aftanas and Golocheikine, 2001). Therefore, it can be inferred that mindfulness training may positively influence the reduction of short-video addiction, and an individual's level of mindfulness may serve as a negative predictor of such addiction.

### 1.2 The mediating role of self-control

Self-control refers to the ability to modify one's responses in accordance with ideals, values, ethics, and social expectations, as well as to sustain the pursuit of long-term goals (Baumeister et al., 2007). The I-PACE model (Brand et al., 2016, 2019), serves as a framework specifically designed to examine irrational behaviors in human interactions with technology. It integrates individual characteristics, emotional and cognitive responses, executive functions, and decision-making processes, thereby providing a robust theoretical foundation for understanding internet addiction behaviors. In this model, self-control is a crucial variable in decision-making execution and is closely associated with addictive behaviors. Studies have demonstrated that the content recommendation strategies employed by short-video applications can leverage artificial intelligence algorithm technology (Xu et al., 2019), continuously suggesting personalized videos to entice users to keep watching. Individuals with poor self-control are more susceptible to excessive use of video streaming services (Hasan et al., 2018) and are particularly prone to overusing short-video applications.

The level of individual mindfulness is closely linked to self-control. A prerequisite for self-control is the individual's ability to recognize their psychological and behavioral states, allowing them to decide whether to regulate these states. Thus, attention control emerges as a crucial component of self-control. Bishop et al. (2004) noted that mindfulness encompasses two primary aspects: attention control and the maintenance of an open and accepting attitude toward current experiences. Therefore, individuals with higher levels of mindfulness are better equipped to focus their attention on their present physical and mental experiences, regulate their attention, and consequently enhance their self-control. Research has confirmed a significant positive correlation between mindfulness and self-control (Bowlin and Baer, 2012). Moreover, the strength model of self-control posits that self-control is a limited and renewable psychological resource that can be depleted, potentially leading to changes in an individual's emotions and behaviors (Baumeister et al., 1998). The more self-control behaviors one engages in, the greater the degree of self-depletion experienced. Previous research has found that brief mindfulness training can enhance an individual's performance on subsequent self-control tasks (Friese et al., 2012). Thus, higher levels of individual mindfulness correspond to improved self-control abilities. Individuals with elevated mindfulness levels are better able to alleviate or recover from the self-depletion resulting from exercising self-control. In addition, neuroimaging studies reveal that the brain regions activated during mindfulness practices and self-control tasks share similarities. Specifically, mindfulness meditation is associated with neural activation in the prefrontal cortex and the cingulate cortex (Gotink et al., 2016), with the prefrontal cortex playing a significant role in the self-control process (D'Esposito et al., 1995).

In the I-PACE model, emotional and cognitive responses are crucial for executive functions, offering valuable clinical insights into the dynamics of short-video addiction. Mindfulness, as a component of emotional and cognitive responses, significantly influences decision-making related to self-control. Existing research has explored the relationships among mindfulness, self-control, and behavioral addiction from various perspectives. Studies have demonstrated that both mindfulness and self-control negatively predict internet addiction, with self-control serving as a mediating factor between mindfulness levels and internet addiction (Song and Park, 2019). Specifically, higher levels of mindfulness correlate with less problematic internet usage, and mindfulness can influence problematic internet use through the mediating role of self-control (Sinha et al., 2020). Furthermore, college students exhibiting higher levels of addiction demonstrate poorer inhibitory control compared to their peers with lower addiction levels (Wang et al., 2015). In conclusion, the essence of mindfulness lies in self-awareness. Elevated levels of self-awareness can enhance individual self-control (Alberts et al., 2011), mitigate the depletion of self-control resources, and ultimately reduce the likelihood of behavioral addiction. Given the parallels between short-video addiction and other behavioral addiction issues, we hypothesize that self-control mediates the relationship between mindfulness levels and short-video addiction.

### 1.3 The current study

While numerous studies have examined the antecedents of problematic short-video viewing behavior, research investigating the impact of short-video usage—particularly regarding how mindfulness and self-control influence short-video addiction among medical students—remains limited. Furthermore, most studies adopt a variable-centered approach, treating short-video addiction as a homogeneous phenomenon. This approach may overlook individual heterogeneity. Latent Profile Analysis (LPA), a statistical method focused on individuals, classifies individuals into different subtypes based on observed indicators, thereby facilitating the exploration and understanding of diversity within groups (Pápay et al., 2013). Previous studies (Pápay et al., 2013) have employed LPA to identify three categories of problematic online gaming behavior among teenagers: high-risk, low-risk, and no-risk problematic online game usage. High-risk players are predominantly male and often exhibit characteristics such as daily gaming time exceeding 5 h. Pan et al. (2024) utilized the same analytical method to categorize short-video addiction among college students into three groups: non-addiction, low-addiction, and high-addiction, indicating the presence of heterogeneity in short-video addiction among college students. Given the individual differences among medical students, employing LPA to identify the types of short-video addiction within this population is justified.

**Figure 1:**
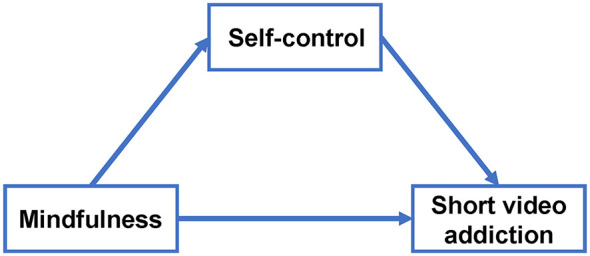
Conceptual mode of research.

This study aims to explore the potential mediating role of self-control in the relationship between mindfulness and short-video addiction in medical students. The research design is presented in Figure 1. We hypothesize: (1) There is a significant negative correlation between mindfulness and short-video addiction among medical students; (2) Self-control mediates the relationship between mindfulness and short-video addiction. (3) The short-video addiction among medical students demonstrates significant heterogeneity. (4) Various subgroups of medical students exhibit distinct relationships between mindfulness, self-control, and short-video addiction, thereby offering a more precise and personalized interpretation of previous research findings. Furthermore, this study will analyze the effect of demographic variables, such as gender and being an only child, on short-video addiction. Existing research indicates that short-video addiction is more prevalent among female students (Liu et al., 2025), while gaming addiction is significantly higher among male students (Tu et al., 2023). Investigating gender differences in short-video addiction will enhance our understanding of the notable variations in internet behavior tendencies across genders. Additionally, comparing only children with non-only children will shed light on how family background and parenting styles influence self-control and the associated risks of addiction related to short-video usage. In summary, this study seeks to examine the factors and mechanisms underlying short-video addiction, aiming to provide clinically effective intervention strategies for individual mental health, thereby assisting medical students in reducing their dependence on short-video content and restoring a healthy lifestyle and academic focus.

## 2 Methods

### 2.1 Sampling and procedure

Data were collected from three medical universities in Fujian Province, China, during the period from November 2024 to 2025.The study was approved by the Medical Ethics Committee of Fujian Medical University (approval number 2024-190). A stratified convenience sampling method was utilized to recruit participants across various academic levels. The inclusion criteria specified that participants must be (1) undergraduate students majoring in medicine, (2) aged between 18 and 25 years, and (3) willing to provide informed consent. The exclusion criteria were defined as (1) an inability to complete the questionnaire in Chinese and (2) prior participation in a similar research study within the preceding 6 months. Data collection was facilitated via the Questionnaire Star platform. The questionnaire took approximately 5 min to complete. The survey was administered in an online group format, either in classroom settings or during breaks, with the assistance of counselors or teachers in small group settings. Importantly, no identifying information from the students was collected. Prior to participation, detailed explanations were provided to the participants, emphasizing that the results would remain strictly confidential and be used solely for scientific research purposes. Participants were also informed of their right to withdraw from the study at any time. All participants agreed to participate in this study and gave informed consent. A total of 1,051 questionnaires were collected. Participants with patterned responses (i.e., 80% or more of the questions choosing the same option) and those with short completion time (defined as <1 min) were excluded. The valid questionnaires were 892, with a response rate of 84.9%. The data integrity check showed no missing values, so missing value analysis was not undertaken.

### 2.2 Measures

#### 2.2.1 The short video addiction scale

This research employed the Short Video Addiction scale (SVA), which was adapted from Leung's Mobile Phone Addiction Index (Qin et al., 2019). And it also included the diagnostic criteria for internet addiction developed by Young based on the standards of pathological gambling (Young, 1998). These standards have been widely used by scholars worldwide to measure the degree of internet addiction among college students and have undergone reliability and validity tests (Ding et al., 2024). The scale consists of 14 items and is divided into 4 dimensions: loss of control (4 items, e.g., “You fail to reduce time spent on short videos”), withdrawal (5 items, e.g., “You feel uneasy without short videos”), escapism (3 items, e.g., “You watch short videos when you feel lonely”), and inefficiency (2 items, e.g., “Short videos reduce your productivity”). Each item rated on a 5-point Likert scale, with responses ranging from 1 (completely not applicable) to 5 (completely applicable). The total score ranges from 14 to 70, with a higher score indicating a stronger addiction to short videos. The scale is divided into four dimensions: loss of control, withdrawal, escapism, and inefficiency. In this study, the Cronbach's alpha coefficient of this scale was 0.90, which was similar to the previously reported value (α = 0.91) (Qin et al., 2019).

#### 2.2.2 The mindfulness attention awareness scale

Mindfulness attention awareness was measured using the Chinese version of the Mindfulness Attention Awareness Scale (MAAS) (Chen et al., 2012). This scale consists of 15 items and employs a six-point Likert scale, with “almost always” scored as 1 and “almost never” scored as 6 (all items are scored positively). A higher the total score reflects a greater level of mindfulness. The Cronbach's alpha coefficient of the original scale was 0.89 (Chen et al., 2012). In this study, the Cronbach's alpha coefficient of this scale was 0.87.

#### 2.2.3 The self-control scale

The self-control ability was measured using the Chinese version of the Self-Control Scale (SCS) for college students (Tan and Guo, 2008). This scale includes 19 items, with 1 point assigned for “completely disagree” and 5 points for “completely agree” (items 1, 5, 11, and 14 are scored positively, while the remaining items are scored negatively). A higher total score indicates better self-control ability. In this study, the Cronbach alpha coefficient for this scale was 0.87, which was similar to the previously reported value (α = 0.86) (Tan and Guo, 2008).

The study performed a Fornell-Larcker discriminant validity analysis, confirming validity as the square root of the average variance extracted for each dimension exceeded the constructs' correlation coefficients. Despite a high correlation (*r* = 0.84) between the out-of-control and inefficient dimensions, this is acceptable due to their theoretical link in addiction theory and being below the 0.90 collinearity threshold.

### 2.3 Test for common method bias

This study used a questionnaire survey and Harman's single-factor test to check for common method bias. The first factor accounted for 21.10% of the variance, below the 40% threshold, indicating no significant bias. To further control potential bias, the unmeasured latent method factor (ULMC) approach was applied within the structural equation model framework.

### 2.4 Statistic analysis

First, a preliminary analysis was conducted to assess the pattern of missing data. All variables had no missing data. SPSS 26.0 was used to perform descriptive, reliability, validity, common method bias, and correlation analyses for mindfulness awareness, self-control, and short-video addiction. The outliers of the multivariate distribution were detected using Mahalanobis distance (*p* < 0.001). Second, the Process 5.0 plugin assessed the relationship between self-control, mindfulness awareness, and short-video addiction, using demographic data as covariates and 5,000 Bootstrap samples to estimate the 95% confidence interval for the mediating effect. Third, to control the common method variance (CMV) and verify the model directionality, a structural equation model (SEM) was constructed using Mplus 8.10. The parameters were estimated using the robust maximum likelihood method (MLR), and the CMV was controlled through the ULMC. Fourth, sensitivity analysis and robustness test was conducted. Fifth, the latent profile analysis (LPA) of the potential profiles of medical students' short-video addiction was conducted using Mplus 8.10. Cross-logical regression analysis was performed using SPSS 26.0 software on mindfulness awareness ability, self-control, gender, place of origin, being an only child, grade, and addiction characteristics. Finally, based on the potential categories determined by LPA, stratified bootstrap mediation analysis was carried out using SPSS 26.0 and the PROCESS 5.0 plugin to explore the heterogeneity patterns of mindfulness, self-control, and short-video addiction in different risk groups.

## 3 Results

### 3.1 Means, standard deviations, and correlation coefficients of the variables

The results of descriptive statistics and correlation analysis between variables are presented in Table 1. The correlation analysis indicates that SVA is significantly negatively correlated with both MAAS and SCS (ρ = −0.206, *p* < 0.001; ρ = −0.439, *p* < 0.001). Conversely, MAAS and SCS exhibit a significant positive correlation (ρ = 0.345, *p* < 0.001).

**Table 1:** Means, standard deviations, and correlation coefficients of the variables.

Variable	*N* (%)	Mean (SD)	SVA	MAAS	SCS
Gender *n* (%)					
Female	633 (71.0)				
Male	259 (29.0)				
Grade *n* (%)					
Freshmen	263 (29.5)				
Sophomores	306 (34.3)				
Juniors	228 (25.6)				
Senior	95 (10.7)				
Student's place of origin					
urban	453 (50.8)				
Rural	439 (49.2)				
Only child *n* (%)					
Yes	225 (25.2)				
No	667 (74.8)				
**Overall score of SVA**		40.29 (10.98)		−0.206^***^	−0.439^***^
Loss of control		11.67 (2.97)			
Withdrawal		12.72 (4.75)			
Escapism		9.36 (3.35)			
Inefficiency		6.54 (2.26)			
**MAAS**		54.93 (11.23)	−0.206^***^		0.345^***^
**SCS**		58.75 (10.71)	−0.439^***^	0.345^***^	

^***^indicates P <0.001. Spearman correlation analysis was employed.

MAAS, Mindful Attention Awareness Scale; SCS, Self-control Scale; SVA, short-form video application addiction.

### 3.2 Testing for the mediation model

Before the main analysis, we assessed the regression model's assumptions. The Kolmogorov-Smirnov test showed some variables deviated from normality (*p* < 0.05), but Q-Q plots indicated only moderate deviation. Variance inflation factors (VIF) were all below 3.0 (range: 1.15–1.16), suggesting no severe multicollinearity. Three multivariate outliers were found using Mahalanobis distance (*p* < 0.001) and were kept in the analysis. A sensitivity check excluding these outliers confirmed the results' stability (Supplementary Table 1).

**Figure 2:**
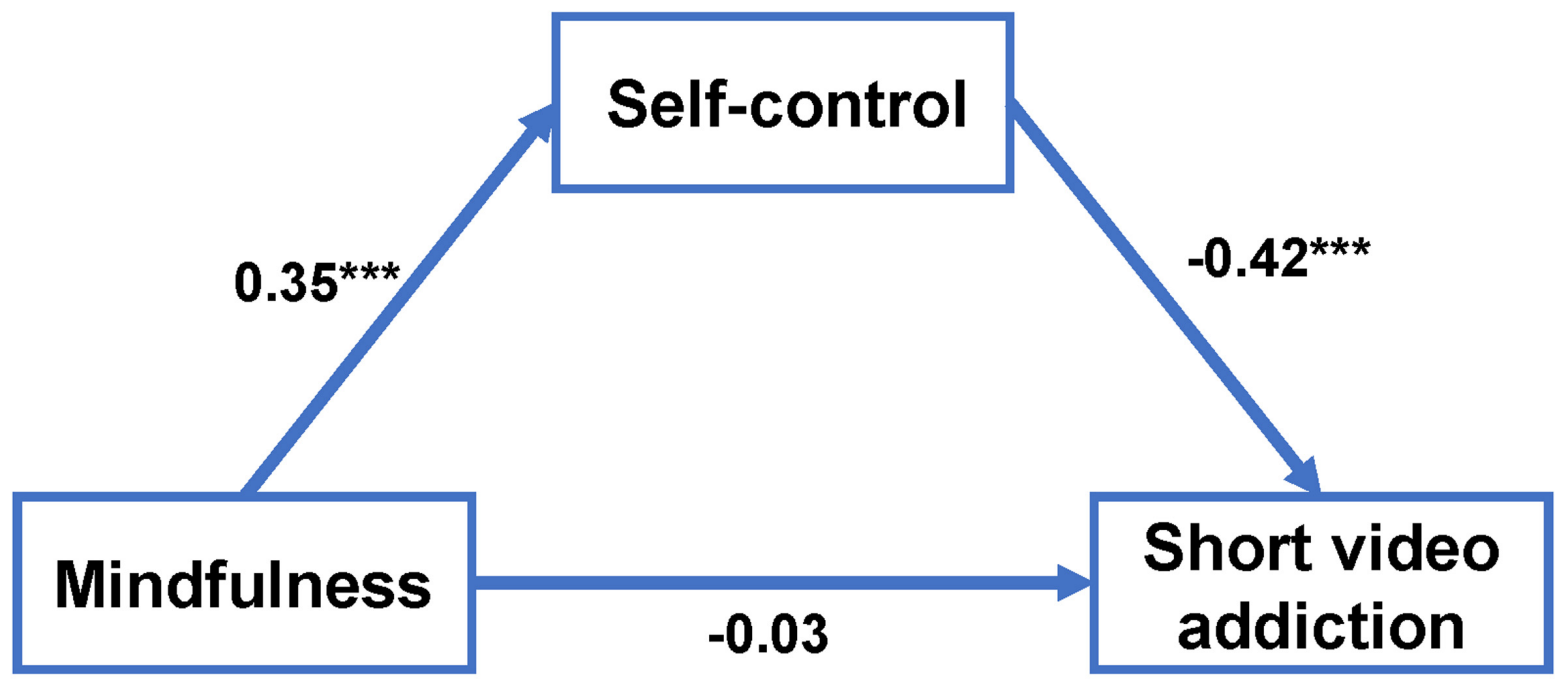
The path coefficients in the mediation model. ***P < 0.001.

Using the Model 4 in the Process 5.0 plugin of SPSS, the mediation analysis was conducted while controlling all demographic variables. All variables were standardized, the results are shown in Table 2. There is a significant negative relationship between MAAS and SVA (β = −0.18, *p* < 0.001), and a significant positive relationship between MAAS and SCS (β = 0.35, *p* < 0.001). Both MAAS and SCS, respectively had a negative correlation with SVA (β = −0.03, *p* > 0.05; β = −0.42, *p* < 0.001). The results are presented in Figure 2. The mediating effect was verified using the Bootstrapping method, with results presented in Table 3. The 95% confidence interval from the Bootstrap analysis does not encompass the value of 0, which indicates that the mediating effect is statistically significant. Furthermore, the total indirect effect is significant, representing 83.33% of the total effect.

**Table 2:** Testing for mediation effect of MAAS on SVA.

Predictor	Model 1 (SVA) Dependent variable			Model 2 (SCS) Mediatory variable			Model 3 (SVA) Dependent variable		
	β	*t*	Boot 95% CI	β	*t*	Boot 95% CI	β	*t*	Boot 95% CI
Gender	0.05	0.72	−0.09, 0.20	0.01	0.08	−0.13, 0.14	0.06	0.82	−0.08, 0.19
Grade	0.03	1.02	−0.03, 0.10	−0.03	−0.80	−0.09, 0.04	0.02	0.76	−0.04, 0.08
Student's place of origin	0.15	2.17	0.01, 0.29	−0.10	−1.46	−0.23, 0.03	0.11	1.72	−0.02, 0.23
Only child	0.17	2.05	0.01, 0.32	0.10	1.28	−0.05, 0.25	0.21	2.80	0.06, 0.35
MAAS	−0.18^***^	−5.63	−0.25, −0.12	0.35^***^	11.29	0.29, 0.42	−0.03	−1.08	−0.10, 0.03
SCS							−0.42^***^	−13.18	−0.49, −0.36
*R^2^*	0.05			0.13			0.21		
*F*	9.83^***^			26.27^***^			38.75^***^		

N = 892.

CI, Confidence Interval; MAAS, Mindful Attention Awareness Scale; SCS, Self-control Scale; SVA, short-form video application addiction.

^***^indicates P <0.001.

**Table 3:** The bootstrapping analysis of the mediating effects.

Type	Effect	*SE*	Boot 95% CI	Proportion
Total effect	−0.18	0.03	−0.25, −0.12	
Direct effect	−0.03	0.03	−0.10, 0.03	16.67%
Indirect effect	−0.15	0.02	−0.19, −0.11	83.33%

CI, Confidence Interval.

To ensure the main findings were robust, several sensitivity analyses were performed (Supplementary Table 1). First, analyses were repeated without three outliers, showing stable path coefficients (maximum β difference = 0.01). Second, using standard instead of robust maximum likelihood methods yielded similar results (maximum β difference <0.01). Third, stratified analyses by gender, grade, student origin, and only-child status showed consistent mediation effects across subgroups (indirect β range: −0.12 to −0.19). Finally, a *post hoc* power analysis with GPower 3.1 confirmed that a sample size of N = 892 had >0.99 power to detect the effect.

### 3.3 Structural equation modeling analysis with common method variance control

A simple mediating model was created, proposing that mindfulness (MAAS) predicts short-video addiction (SVA) both directly and indirectly via self-control (SCS). To address common method bias, the ULMC was applied. The findings indicated a significant negative correlation between CMV and MAAS (*r* = −0.79, *p* < 0.001), but no significant correlation between CMV and SCS (*r* = −0.06, *p* = 0.06) or CMV and SVA (*r* = 0.03, *p* = 0.40). Controlling for CMV did not alter the core variable relationships, indicating that common method bias did not affect the main conclusion.

The fit indices for the structural equation model are as follows: χ^2^(df) = 372.553 (72), *p* < 0.001; RMSEA = 0.068 [90% CI (0.062–0.075)]; CFI = 0.918; TLI = 0.887; SRMR = 0.042. These indices suggest that the model demonstrates an acceptable fit to the data. All estimates were derived using the robust maximum likelihood method (MLR) and controlled for common method variance (CMV). The findings indicate that mindfulness is significantly positively correlated with self-control (β = 0.48, *p* < 0.001), while self-control is significantly negatively correlated with short-video addiction (β = −0.52, *p* < 0.001). The direct effect was not significant [β = −0.01, 95% CI (−0.12, 0.10)], whereas the indirect effect was significant [β = −0.25, 95% CI (−0.32, −0.19)], with the mediating effect accounting for 95.4% of the total effect (Supplementary Tables 2, 3).This study also assessed the directionality of the theoretical model by comparing its goodness-of-fit with a reverse model (short-video addiction → self-control → mindfulness). Both models accounted for gender, grade, only-child status, place of origin, and included the CMV factor. The original model demonstrated a better fit (Δχ^2^ = −9.68) and had lower AIC and BIC values, supporting the proposed theoretical direction (Supplementary Table 4).

### 3.4 The latent profile analysis

**Figure 3:**
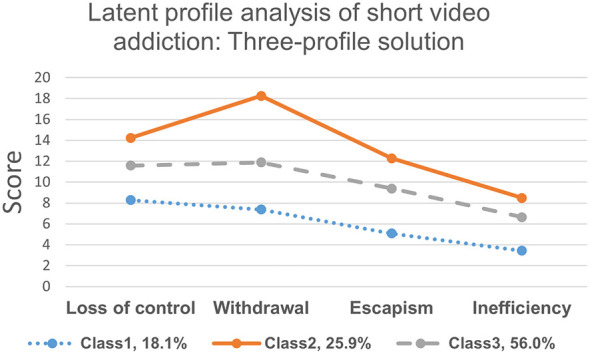
The latent profile analysis of short video addiction: three-profile solution.

Utilizing the four dimensions of short-video addiction (loss of control, withdrawal, escapism, and inefficiency), a heterogeneous subgroup of medical students was identified. Models with potential categories ranging from one to five were estimated, as detailed in Table 4. The information criteria indicated that the Akaike Information Criterion (AIC), adjusted Bayesian Information Criterion (aBIC), and Bayesian Information Criterion (BIC) values decreased with an increasing number of categories. However, the smaller categories in the four-category and five-category models represented only 5.1% and 5.5% of the sample, respectively. The likelihood ratio test demonstrated that the three-category model was significantly superior to the two-category model (LMR-LRT *p* < 0.001, BLRT *p* < 0.001), whereas the enhancement of the five-category model over the four-category model was not statistically significant (LMR-LRT *p* = 0.456). This suggests that further increasing the number of categories did not yield significant improvements in likelihood. Regarding classification accuracy, the three-category model exhibited the highest entropy value, with average posterior probabilities for each category being 0.91, 0.89, and 0.91, respectively. These values exceed the standard threshold of 0.80, indicating a high degree of classification certainty. In light of the information criteria, the significance of the likelihood ratio test, the category proportions (17.6%, 25.3%, 57.1%), and the principle of parsimony, the three-category model was ultimately selected. This model categorized short-video addiction users into low-risk, medium-risk, and high-risk addiction groups. Table 5 shows the model's reliability, with positive predictive values between 88% and 91%. The addiction traits of short videos are derived from the mean values of the short-video addiction scale dimensions. Figure 3 displays the average for each indicator. Profile 1 shows low levels across all indicators and is labeled as low-risk. Profile 2 has the highest average scores in addiction dimensions and is labeled high-risk. Profile 3 indicates medium addiction risk. Additionally, covariance analysis was used to explore the link between personal data classification and achievement goal orientation dimensions, with Table 6 presenting the average values and standard deviations for each goal orientation. A one-way ANOVA revealed significant effects of the three latent categories on the four dimensions of short-video addiction among medical students (*p* < 0.05). Pairwise comparisons showed that C1 scored lower than C3, and C3 scored lower than C2 (*p* < 0.05).

**Table 4:** Model fit indices of the medical college students' short video addiction.

Model	AIC	aBIC	BIC	Entropy	LMR p	BLRT p	Percentage of sample (%)
1-class	18,471.916	18,484.858	18,510.264	—	—	—	100.0
2-class	17,591.209	17,612.238	17,653.524	0.784	<0.001	<0.001	30.6/69.4
3-class	17,225.470	17,254.588	17,311.752	0.785	<0.001	<0.001	17.6/25.3/57.1
4-class	17,133.046	17,170.251	17,243.295	0.777	0.007	<0.001	14.6/5.1/33.4/45.8
5-class	17,062.166	17,107.460	17,196.383	0.762	0.456	<0.001	13.2/30.3/12.2/5.5/38.8

**Table 5:** Most likely latent profile membership (row) by latent profile (column).

Profile	*N*	Percentage	Attribution probability		
			C1	C2	C3
C1	157	17.60	0.912	0.000	0.088
C2	226	25.34	0.000	0.888	0.112
C3	509	57.06	0.036	0.060	0.905

**Table 6:** Comparison of scores in short-form video addiction and various dimensions among different types of medical college students.

Profile	Withdrawal	Escapism	Loss of control	Inefficiency
C1	8.16 ± 2.74	7.24 ± 2.29	4.96 ± 2.36	3.32 ± 1.45
C2	14.35 ± 2.26	18.58 ± 3.06	12.32 ± 1.92	8.58 ± 1.28
C3	11.57 ± 1.97	11.81 ± 2.92	9.40 ± 2.53	6.63 ± 1.60
*F*	368.36^***^	788.79^***^	450.67^***^	572.07^***^
LSD	1 <3 <2	1 <3 <2	1 <3 <2	1 <3 <2

^***^indicates P <0.001.

### 3.5 The relationship between demographic variables, mindfulness, self-control, and short-video addiction

Multiple logistic regression analyses were conducted using the low-risk addiction profile as the reference group, as this profile encompasses individuals with the lowest probability of internet addiction. As illustrated in Table 7, non-only children exhibited higher probabilities of medium and high-risk addiction. Furthermore, the results indicated that medical students with lower self-control abilities were more likely to develop short-video addiction, particularly at medium and high-risk levels.

**Table 7:** Results of the multinominal logistic regression predicting latent profile membership.

Variables	C2 (High risk)		C3 (Medium risk)	
	OR	95% CI	OR	95% CI
Gender (female = 0)	0.944	0.579–1.537	0.724	0.483–1.086
Grade (senior as referent)	0.947	0.752–1.194	0.860	0.710–1.041
Student's place of origin (rural = 0)	0.677	0.419–1.091	0.785	0.525–1.171
Only child (No = 0)	0.449	0.260–0.775	0.637	0.415–0.979
MAAS	0.988	0.967–1.009	0.986	0.969–1.003
SCS	0.877	0.855–0.900	0.953	0.935–0.972

OR, odds ratio; CI, Confidence Interval.

### 3.6 Differences in mediation effects across latent profile classes

Hierarchical Bootstrap mediation analysis revealed different patterns of the mindfulness and short-video addiction relationship across these categories. In the low-risk group, neither total nor indirect effects were significant. In the medium-risk group, the indirect effect was significant [β = −0.04, 95% CI (−0.07, −0.03)], but the direct effect was not. Similarly, in the high-risk group, the indirect effect was significant [β = −0.04, 95% CI (−0.07, −0.02)], with no significant direct effect (Table 8).

**Table 8:** Differences in mediation effects across latent profile classes.

Profile	*N*	Total effect β [Boot 95% CI]	Direct effectβ [Boot 95% CI]	Indirect effect β [Boot 95% CI]
**Class1**	157	0.01 [−0.05, 0.07]	0.02 [−0.04, 0.08]	−0.006 [−0.03, 0.02]
**Class2**	226	−0.02 [−0.08, 0.05]	0.02 [−0.04, 0.09]	−0.04 [−0.07, −0.02]
**Class3**	509	−0.08 [−0.12, −0.04]	−0.04 [−0.08, 0.01]	−0.04 [−0.07, −0.03]

CI, Confidence Interval.

## 4 Discussion

This study focused on medical undergraduate students and examined the impact of mindfulness awareness on short-video addiction. The results were verified through the mediating role of self-control, which partially supported the hypothesis. Specifically, there was a negative association between an individual's mindfulness and the short-video addiction. Self-control played a complete mediating role between mindfulness and short-video addiction.

### 4.1 Short-video addiction profiles

In this study, the proportion of medical students exhibiting addiction to short videos was found to be 37.11%, significantly higher than the 23.57% reported in a prior study involving college students (Wu et al., 2024). This finding underscores that short video addiction has emerged as a critical issue, posing a substantial threat to the mental health of medical students. Consequently, it warrants the attention of educational authorities, academic institutions, families, and society at large. Previous research has indicated that medical students experience elevated levels of stress and are susceptible to behavioral addiction problems (Hakami et al., 2021; Li et al., 2022). Short videos are characterized by higher information density and lower participation costs compared to traditional social media platforms, making it imperative to thoroughly understand the motivations behind excessive use of short video applications and the underlying behavioral patterns. This study utilized latent profile analysis to explore the potential patterns of short video addiction. Consistent with previous studies (Liu et al., 2025), this research identified distinct patterns of short video addiction among Chinese medical students. Specifically, three characteristic models were established: low-risk, medium-risk, and high-risk characteristics. This further corroborates the heterogeneity of short video addiction among medical students, allowing for an analysis of key influencing factors across different subtypes.

Multivariate logistic regression analysis indicates that, compared to only children, non-only children exhibit a higher rate of moderate to high-risk short-video addiction. Non-only children may have a greater need for social interaction due to sibling interactions, making short-video platforms one of the tools for extending social engagement. Moreover, the family systems theory model posits that each subsystem within the family is critically important, and these subsystems interact and influence one another (Cox, 2010). The interaction patterns among family members shape an individual's psychological state and behavioral tendencies. Existing studies have found that among adolescents with internet addiction, neglectful families represent a relatively high proportion, with children often perceiving their parents' emotional availability as lower (Karaer and Akdemir, 2019). Therefore, the attention of parents in multi-child families may be diluted, leading to non-only children potentially having limited resources for companionship or family responsibilities. As a result, they may turn to online short-video content to seek compensation and self-comfort to satisfy their emotional needs (Zhan and Zhu, 2025). In conclusion, this suggests that when examining the differences between only children and non-only children, it is essential to consider variations in family education and parenting styles. Future research should further delineate specific mechanisms. In this study, we found no significant differences in the risk of short-video addiction (SVA) based on gender, grade, or the student's place of origin, aligning with previous research (Wu et al., 2024). Although multiple studies have indicated that the degree of SVA tends to be higher among females (Liu et al., 2025; Zhao et al., 2024), the observed discrepancy may stem from the diverse content and platforms of short videos, which attract a broad spectrum of users and potentially obscure the effects of demographic factors on usage behavior. Furthermore, the grade of college students and their place of origin did not significantly influence the risk of short-video addiction. This lack of differentiation may be attributed to the widespread availability and affordability of smart communication tools, which allow equitable access to educational and informational resources, thereby mitigating potential disparities. Conversely, certain individual psychological and social factors, such as personality traits (Zhang et al., 2024), may exert a more direct influence on SVA than demographic variables like gender, grade, or place of origin.

### 4.2 The association between self-control and short-video addiction

The research findings demonstrate that self-control plays a significant mediating role between individual mindfulness awareness ability and SVA (β = −0.42, *p* < 0.001), thereby supporting the H2 hypothesis. Specifically, there is a negative correlation between self-control and SVA. Self-control acts as a mediator between mindfulness awareness ability and SVA. This observation aligns with previous studies (Li et al., 2021a; Liu et al., 2018) and underscores the critical importance of incorporating self-control enhancement in intervention strategies for addressing short-video addiction. Individuals with diminished self-control exhibit lower resistance to novel stimuli and, when confronted with the diverse content of short videos, find it challenging to resist, leading to addiction. Furthermore, these individuals often struggle with time perception and management, resulting in distorted time perception during short-video consumption, which complicates their ability to disengage (Zhu et al., 2021). As shown in this study, the medical students in the medium-risk addiction group and the high-risk addiction group scored higher than those in the low-risk addiction group in all four dimensions of short-video addiction, especially in the withdrawal dimension and the loss-of-control dimension. Additionally, a vicious cycle exists between poor self-control and SVA, as the stimulating and content-rich nature of short videos captures individuals' attention with minimal cognitive effort. Long-term viewing stimulates the lower regions of the cerebral cortex, particularly those associated with emotion processing, while inhibiting the activities of the higher regions responsible for self-control and attention. This pattern may lead to a decline in self-control abilities, further increasing an individual's susceptibility to SVA (Yan et al., 2024).

### 4.3 The mediating role of self-control

Our research findings indicate a negative correlation between individual mindfulness awareness and short-video addiction (SVA). However, when the mediating variable of self-control is introduced, the negative impact of mindfulness on SVA becomes non-significant. This suggests that the negative influence of mindfulness on SVA is entirely mediated by self-control. This finding further underscores the complex and interrelated structural components of mindfulness and self-control, as both constructs encompass the element of attention (Kabat-Zinn, 2003; Harwood et al., 2023). Derryberry and Reed proposed that effective attention control requires not only the active utilization of cognitive resources to focus on goal-relevant information but also the suppression of irrelevant information (Derryberry and Reed, 2002). Mindfulness primarily enhances self-regulation through the processes of attention, awareness, and acceptance (Buric, 2025), which in turn strengthen the conflict monitoring function of the anterior cingulate cortex and improve executive control capabilities (Lindsay and Creswell, 2017). Consequently, the effective negative impact of mindfulness on short-video addiction likely necessitates the mediating role of self-control. In other words, the attention and awareness skills fostered by mindfulness may need to be converted into the actual ability to suppress impulses and regulate behaviors to effectively mitigate the risk of addiction.

This fully mediating model, however, reveals significant heterogeneity among various groups at risk of addiction. Our findings suggest that the mediating effect of self-control was significant only in the medium-risk and high-risk addiction groups, but not in the low-risk group. Notably, the medium-risk group demonstrated the most pronounced indirect effect [β = −0.04, 95% CI (−0.07, −0.03)] as well as the strongest total effect (β = −0.08). We propose that this observed heterogeneity can be elucidated by the I-PACE model (Brand et al., 2019). According to the I-PACE model, the progression of addictive behaviors involves a transition from an early stage to a later stage (Brand et al., 2019). During the early stage, behaviors may primarily be driven by gratification, with the ventral striatum and dorsolateral prefrontal cortex playing pivotal roles. At this time, the emotions and cognitive processes involved in decision-making for specific behaviors are primarily regulated by general inhibitory control and self-regulation/self-oriented regulation. However, general inhibitory control has already shown signs of decline (Brand et al., 2019). In the later stages, compensatory effects become predominant, and behaviors gradually become habitual, with the dorsal striatum assuming a more prominent role. At this point, the regulation of behavior by general inhibitory mechanisms may become more challenging, and stimulus-specific inhibitory control also exhibits deficiencies. Consequently, decision-making for specific behaviors may be increasingly influenced by impulsivity and reactive responses (Brand et al., 2019). We therefore hypothesize that individuals in the low-risk addiction group may possess relatively adequate self-control resources (Baumeister et al., 2007) and have not yet developed problematic patterns of short video usage. Furthermore, mindfulness may exhibit a ceiling effect on the potential enhancement of self-control resources through the monitoring-acceptance mechanism, rendering both direct and indirect effects insignificant. In contrast, individuals in the medium-risk addiction group might be in the early stages of addiction, where goal-oriented systems still hold sway (Lei et al., 2025; Daw et al., 2005). At this stage, mindfulness can enhance self-regulation through attention, awareness, and acceptance, improve proactive control (Li et al., 2018), and help individuals make purposeful responses aligned with long-term values (Buric, 2025), reducing the risk of addiction. In the high-risk addiction group, despite a significant indirect effect, the total effect is not significant (β = −0.02, *p* > 0.05), indicating that these individuals may have reached a later stage where compensatory effects outweigh gratification, and behavior has become habitual. Even though they might attempt to resist impulses through inhibitory control, the entrenched habits and depleted self-control resources make it challenging. On the other hand, prolonged exposure to highly stimulating content offers users immediate emotional gratification (Jiang et al., 2024) but also induces frequent shifts in attentional focus (Christakis et al., 2004), thereby impairing the capacity for sustained goal-directed behavior. Consequently, given the entrenched nature of habitual systems and the depletion of chronic self-control resources, merely enhancing cognitive control may prove insufficient to fully counteract behavioral inertia, leading to a diminished overall effect. This observed group difference indicates the necessity for tailored intervention strategies for distinct risk groups. For medical students exhibiting moderate-risk short-video addiction, prioritizing mindfulness training may facilitate self-regulation and enhance proactive control. In contrast, for high-risk addiction groups, where behaviors have become habituation, a simple mindfulness intervention may not suffice to counteract automatic usage patterns. It may be essential to integrate cognitive behavioral therapy to enable individuals to “first distance themselves from their habitual responses,” thereby achieving a more profound and sustained improvement in addiction (Liang et al., 2025; Stevens et al., 2019).

## 5 Implication and limitation

The research conclusions have significant value for both the theoretical and practical aspects of short-video addiction. This study found that the self-control might be a key factor in reducing the risk of short-video addiction among medical students. Mindfulness training can indirectly reduce the tendency of short-video addiction by enhancing an individual's self-control resources, especially for those with medium-risk short-video addiction. This discovery provides psychological scientific basis for educational departments and universities to prevent and intervene in students' addiction behaviors. Medical courses can include mindfulness training, for example, explicitly teaching mindfulness awareness, aiming to help medical students develop the more important ability to control their own thoughts and actions. Theoretically, this study provides a basis for researching the psychological factors of SFV addiction and reveals how mindfulness affects SFV addiction through the self-control mechanism. This study also has some limitations. Firstly, we employed a cross-sectional design to evaluate our hypothesized model. This design cannot determine the causal relationship between variables. Apart from the path proposed by this study, other theoretical directions also have possibilities. For instance, addiction to short videos may lead to a decrease in an individual's mindfulness level and depletion of self-control, or self-control, as a fundamental cognitive trait, may also pre-emptively affect the effectiveness of an individual's mindfulness training and the addictive tendency of using short videos. Thus, future research can adopt a longitudinal research design to further explore the dynamic relationship between mindfulness, self-control and short-video addiction. The cross-lagged panel design or the random intercept cross-lagged model can be used to verify the causal direction and the directionality of the mediating effects, in order to more clearly reveal the dynamic mechanism of short-video addiction. Secondly, we collected data using self-report tools, and social expectation bias may affect the participants' responses. Moreover, this study used convenience sampling, which may not represent a broader group of medical students. The generalizability of the research conclusions is limited, and the conclusions should be viewed with caution. Future research can adopt random sampling or stratified sampling methods to enhance the generalizability of the research results. Finally, the research design failed to fully incorporate the influence of personality traits and other psychological factors on short-video addiction. Future research can combine the I-PACE model (Brand et al., 2019) and integrate personality variables (Wu et al., 2024), emotional cognitive responses, and executive functions (Alloway and Alloway, 2012; Toh et al., 2023) to provide a more comprehensive explanation of the intrinsic mechanism of short-video addiction.

## 6 Conclusion

Short-video addiction is a pathological factor that adversely affects the academic performance and personal development of college students. In the context of short-video addiction, mindfulness awareness and self-control are crucial personal resources. The results of this study indicate that medical students with higher levels of mindfulness awareness possess stronger self-control resources that help them resist short-video addiction. Furthermore, the findings reveal that mindfulness has a negative association with short-video addiction, while self-control mediates the relationship between mindfulness and short-video addiction. These results contribute new evidence to the existing literature on the relationship between mindfulness and behavioral addiction.

## Data Availability

The original contributions presented in the study are included in the article/Supplementary material, further inquiries can be directed to the corresponding authors.
