# Supplementary material for: The relationship between mindfulness, self-control, and short video addiction among medical students
Source: Front Psychol. 2026 May 1;17:1746374. doi: 10.3389/fpsyg.2026.1746374 (PMC13177302; doi:10.3389/fpsyg.2026.1746374)
Supplement: Supplementary file 1 [file Table_1.docx]

**Supplementary Table1. Sensitivity Analyses of the Mediating Effect**

| **Analysis condition** | **N** | **Total effect**  **β [Boot 95%CI]** | **Direct effect**  **β [Boot 95%CI]** | **Indirect effect**  **β [Boot 95%CI]** |
| --- | --- | --- | --- | --- |
| Main analysis(full sample) | 892 | -0.18 [-0.25, -0.12] | -0.03 [-0.10, 0.03] | -0.15 [ -0.19. -0.11] |
| Excluding outliers | 889 | -0.22 [-0.28, -0.15] | -0.06 [-0.12, 0.00] | -0.16 [-0.20, -0.12] |
| **Gender stratification** |  |  |  |  |
| Male | 259 | -0.15[-0.27, -0.03] | -0.03 [-0.15, 0.08] | -0.12[-0.21, -0.06] |
| Female | 633 | -0.20 [-0.28, -0.13] | -0.04 [-0.11, 0.04] | -0.17 [-0.22,-0.12] |
| **Grade stratification** |  |  |  |  |
| Lower grade | 569 | -0.14 [-0.22,-0.06] | -0.15 [-0.09, 0.07] | -0.13 [-0.18, -0.09] |
| Higher grade | 323 | -0.25 [-0.36, -0.14] | -0.06 [-0.17,0.04] | -0.19 [-0.27, -0.12] |
| **Student’s place of**  **Origin stratification** |  |  |  |  |
| Rural | 439 | -0.19 [-0.28, -0.10] | -0.05 [-0.14, 0.04] | -0.14 [-0.20,-0.09] |
| Urban | 453 | -0.18 [-0.27,-0.09] | -0.03 [-0.11,0.07] | -0.16 [-0.22, -0.10] |
| **Only child** |  |  |  |  |
| No | 667 | -0.19 [-0.26, -0.11] | -0.03 [-0.11, 0.04] | -0.15 [-0.20, -0.11] |
| Yes | 225 | -0.18 [-0.30, -0.05] | -0.03 [-0.17, 0.10] | -0.14 [-0.24, -0.08] |

N=892. CI, represents Confidence Interval;

MAAS, Mindful Attention Awareness Scale; SCS, Self-control Scale; SVA, short-form video application addiction.

**Supplementary Table2. Path Coefficients in the Structural Equation Model**

| **Predictor** | **SCS**  **Mediatory variable** | | | **SVA**  **Dependent variable** | | |
| --- | --- | --- | --- | --- | --- | --- |
|  | *β* | *SE* | 95%CI | *β* | *SE* | 95%CI |
| **Gender** | -0.001 | 0.04 | -0.07 0.07 | 0.02 | 0.04 | -0.06, 0.09 |
| **Grade** | -0.01 | 0.03 | -0.07, 0.05 | -0.003 | 0.03 | -0.07, 0.06 |
| **Student’s place of**  **origin** | 0.04 | 0.03 | -0.03, 0.10 | -0.07* | 0.04 | -0.14, -0.003 |
| **Only child** | -0.06 | 0.04 | -0.13, 0.01 | -0.10** | 0.04 | -0.18, -0.03 |
| **MAAS** | 0.48*** | 0.05 | 0.39, 0.57 | -0.01 | 0.06 | -0.12, 0.10 |
| **SCS** | - | - | - | -0.52*** | 0.05 | -0.61, -0.43 |
| ***R^2^*** | 0.24 |  |  | 0.29 |  |  |

N=892; CI = confidence interval based on robust standard errors (MLR estimation);

* p < 0.05, ** p < 0.01, *** p < 0.001

MAAS, Mindful Attention Awareness Scale; SCS, Self-control Scale; SVA, short-form video application addiction.

**Supplementary Table3. The SEM analysis of the mediating effects**

| **Type** | **β** | **SE** | **Z-value** | **P-value** | **95%CI** | **Proportion** |
| --- | --- | --- | --- | --- | --- | --- |
| Total effect | -0.26 | 0.05 | -4.92 | ＜0.001 | -0.37, -0.16 | 100% |
| Direct effect | -0.01 | 0.06 | -0.22 | 0.83 | -0.12, 0.10 | 4.56% |
| Indirect effect | -0.25 | 0.03 | -7.67 | ＜0.001 | -0.32, -0.19 | 95.44% |

**Supplementary Table4. Comparison of fitting indicators between the original model and the reverse model**

|  | Original model  Mindfulness→ Self Control→ Addiction | Reverse model  Addiction→Self Control→Mindfulness |
| --- | --- | --- |
| χ²(df) | 372.55 (72)*** | 382.23 (72)*** |
| P-value | <0.001 | <0.001 |
| RMSEA [90%CI] | 0.068 [0.062, 0.075] | 0.070 [0.063, 0.076] |
| CFI | 0.918 | 0.915 |
| TLI | 0.887 | 0.883 |
| SRMR | 0.042 | 0.048 |
| AIC | 20577.78 | 20588.20 |
| BIC | 20812.66 | 20823.08 |

***means P < 0.001；

RMSEA, Root Mean Square Error of Approximation；CFI, Comparative fit index；TLI, Non-Normed Fit Index；SRMR, standardized root mean square residual；AIC, Akaike information criterion；BIC, bayesian information criterionΔχ², the difference between the chi-square value of the two models
